# TGFβ1 in fibroblasts-derived exosomes promotes epithelial-mesenchymal transition of ovarian cancer cells

**DOI:** 10.18632/oncotarget.21635

**Published:** 2017-10-06

**Authors:** Wenqian Li, Xiaoxue Zhang, Ji Wang, Mengchen Li, Canhui Cao, Jiahong Tan, Ding Ma, Qinglei Gao

**Affiliations:** ^1^ Cancer Biology Research Center (Key Laboratory of The Ministry of Education), Tongji Hospital, Tongji Medical College, Huazhong University of Science and Technology, Wuhan, Hubei 430030, People's Republic of China

**Keywords:** ovarian cancer, CAF, exosomes, TGFβ1, epithelial-mesenchymal transition

## Abstract

Cancer-associated fibroblasts (CAF), a major component of the tumor microenvironment, play an important role in interacting with neoplastic cells to promote ovarian cancer progression. Exosomes are nano-sized vesicles that mediate the cross-talk between different cell types. An increasing number of studies have focused on the fact that tumor cell-derived exosomes influence stromal cells. However, the mechanism by which CAF-derived exosomes modulate cancer cells in ovarian cancer remains obscure. To investigate the role of CAF exosomes in ovarian cancer, we examined the exosomal content of paired primary, metastatic and normal fibroblasts from seven stage IIIC ovarian cancer patients by ELISA. We found that in ovarian CAF-derived exosomes, TGFβ1 was upregulated compared to normal omentum fibroblasts (NOF). Exosomes derived from CAF were taken up by ovarian SKOV-3 and CAOV-3 cell lines during co-culture and induced malignant behaviors in cancer cells, including an enhanced migration and invasion ability and the promotion of epithelial-mesenchymal transition (EMT) by activating the SMAD signaling pathway. Our results indicate that the role of TGFβ1 in CAF exosomes triggers ovarian cancer cells into a more aggressive phenotype, suggesting that targeting CAF exosomes could be a potential treatment in ovarian cancer.

## INTRODUCTION

Epithelial ovarian cancer, one of the leading causes of death in women, is generally characterized by widespread peritoneal metastasis and a poor 5-year survival rate [1, 2]. Of all the organs within the abdominal cavity, the omentum is the most notable site of ovarian cancer metastasis [3]. The tissue-specific metastasis or pre-metastatic niches, usually referred to as “seed and soil”, represent a specialized microenvironment that is favorable for tumor cells to survive and grow [4, 5]. During tumorigenesis and metastasis, cancer cells interact with the surrounding environment, which is mainly composed of fibroblasts, and transform normal fibroblasts into an active cancer-associated fibroblasts (CAF) [6, 7]. Previous studies suggest that CAF play a pivotal role in establishing a metastatic niche and promoting tumor cell proliferation, invasion and metastasis by secreting chemokines and cytokines in this environment [8–10]. However, it is still unclear how fibroblasts reprogram tumor cells and how they contribute to omentum metastasis at early ovarian cancer progression.

Exosomes are 30-150 nm bilayer membrane vesicles that are released by diverse types of living cells [11, 12]. They are formed by the inward budding of the plasma membrane and act as mediators in intercellular communication by delivering protein, microRNA or mRNA from donor cells to recipient cells [13]. Increasing evidence reveals that exosomes isolated from CAF influence neoplastic epithelial cells in tumor growth, epithelial-mesenchymal transition (EMT), drug resistance, and metastasis [7, 14–18]. Thus, the mechanism of cell-cell crosstalk via exosomes, particularly the communication between the metastatic niche and the disseminated tumor cells to create intraperitoneal implantations, is pronounced for cancer metastasis.

The formation of metastatic outgrowths requires primary tumor cells to invade through the basement membrane barrier and then disseminate at a distance. During this process, epithelial cells lose polarity and cell-cell adhesion, gaining migratory and invasive properties induced by EMT [19].

Transforming growth factor beta (TGFβ) isoforms participate in a variety of reproductive, differentiation and development programs [20]. Several studies suggest that the TGFβ superfamily is involved in ovarian cancer progression. TGFβ1 and TGFβ2 can either act as a tumor promoter in diverse cancer models or as a tumor suppressor by inhibiting cell proliferation [21, 22]. Although the role of TGFβ in ovarian cancer has been studied extensively, the function of TGFβ1 and TGFβ2 in exosomes remains to be explored to understand how these cytokines contribute to EMT in ovarian cancer. Matrix metalloproteinases (MMPs), especially MMP-2 and MMP-9, are proteases that are capable of degrading the extracellular matrix to support cancer cell escape from the primary tumor site and metastasis in ovarian cancer [23–25]. Recent studies report that tumor cell-derived exosomes promote an EMT phenotype through MMPs and TGFβ as cargo components [26, 27]. Moreover, in addition to its role as a biomarker for ovarian cancer, the biological function of CA-125 in ovarian fibroblasts is rarely known [28]. However, given that the role of fibroblasts during ovarian cancer metastasis is not clarified, the mechanism by which exosomes influence recipient cells by delivering contents remains vague. Thus, it is urgent to discover whether exosomes regulate the communication between tumor cells and fibroblasts to promote EMT in ovarian cancer.

In this study, we investigated whether the release of CAF exosomes and their contained proteins is involved in inducing an EMT phenotype in cancer cells and thus promoting peritoneal metastasis in ovarian cancer. From matching fibroblasts, we observed that exosomes containing TGFβ1 from CAF induced changes in two ovarian epithelial cell lines to a more malignant phenotype via SMAD signaling. Thus, the CAF-derived exosomes may be a potential target for stroma-oriented therapy in ovarian cancer.

## RESULTS

### Isolation and identification of fibroblast-derived exosomes

**Figure 1:**
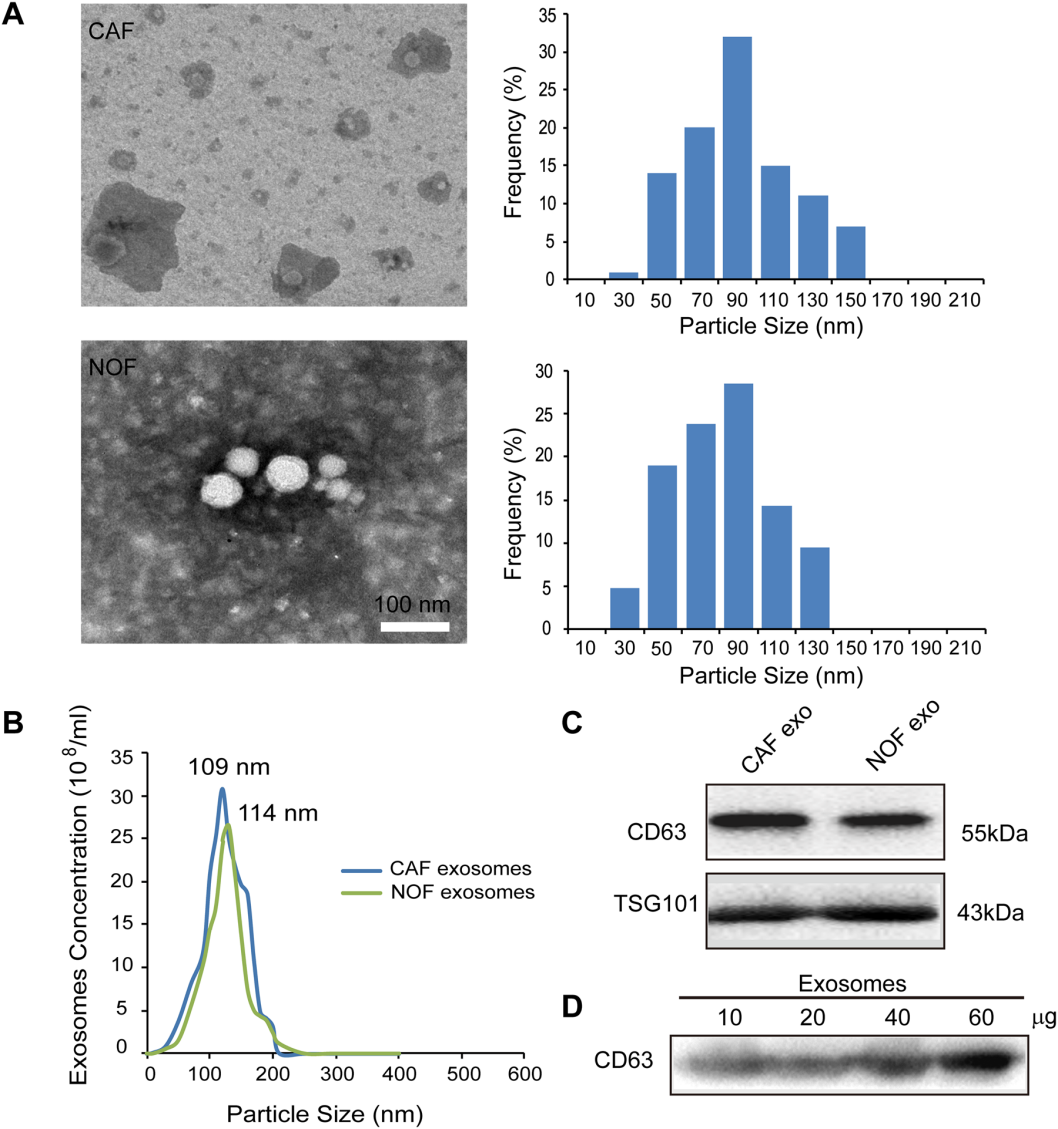
Characterization of exosomes derived from primary stromal fibroblasts **(A)** Transmission electron microscopy images of ovarian CAF-derived exosomes and NOF-derived exosomes with the exosomes diameter distribution represented in histogram. **(B)** NanoSight measurement of particle size distribution and concentration in exosomes. **(C)** Representative images of western blot for the indicated CD63 and TSG101 proteins in fibroblast-derived exosomes lysates. **(D)** CD63 was present in exosomes derived from patient fibroblasts at different exosomal protein concentrations.

Exosomes from CAF and NOF were isolated by ultracentrifugation and were analyzed by transmission electron microscopy (TEM). The distribution of exosome particles was between 30-150 nm (Figure 1A). The exosome size and concentration quantification from the CAF and NOF were analyzed by a NanoSight assay, demonstrating peak sizes at 109 and 114 nm, respectively (Figure 1B). The identification of exosomes was confirmed by markers of CD63 and TSG101 by immunoblotting (Figure 1C). Figure 1D shows that CD63 was concentration-dependent in the fibroblast-derived exosomes.

### Fibroblast-derived exosomes enter and stimulate the migration and invasion of ovarian cancer cells

**Figure 2:**
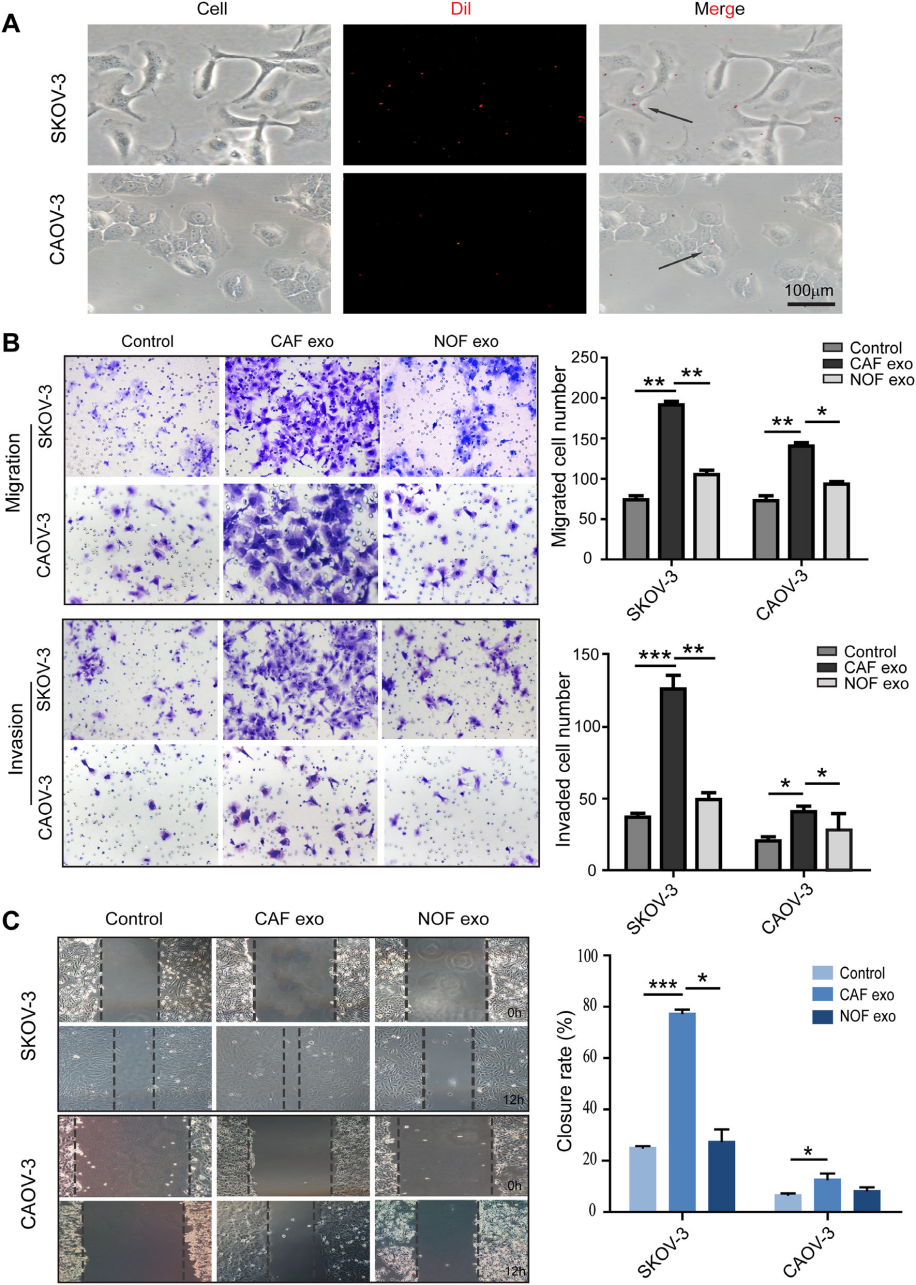
Fibroblast-derived exosomes enter and stimulate migration and invasion of ovarian epithelial cell lines **(A)** Exosomes uptake experiment. SKOV-3 and CAOV-3 cells were cocultured with Dil labeled CAF-derived exosomes for 6h. **(B)** The migration and invasion ability of exosome-treated SKOV-3 and CAOV-3 cells were determined using the Transwell assay. Patients CAF-derived exosomes induced significantly more migration ability and invasiveness than NOF-derived exosomes. Representative images were showed on the left (magnification, ×200), data analysis represented on the right. **(C)** Analysis of tumor cells migration by scratch assay, wound closure rate represent at least three experiments. ^*^ p<0.05, ^**^ p<0.01, ^***^ p<0.001.

To investigate the effects of ovarian fibroblast-derived exosomes on cancer cells, the exosomes from CAF-p, CAF-m and NOF were collected and incubated with the SKOV-3 and CAOV-3 cell lines. We first used fluorescence microscopy to confirm that fibroblast-derived exosomes were taken up by ovarian cancer cells after 6 h of co-culture (Figure 2A). Cell migration and invasion were measured in both cell lines by a Transwell assay. The CAF exosome-treated groups had enhanced migration and invasion abilities compared with the NOF exosome and control groups (Figure 2B and 2C), suggesting that the proteins contained in the CAF exosomes stimulated the SKOV-3 and CAOV-3 cell lines into a more metastatic status.

### TGFβ1 is upregulated in CAF exosomes

**Figure 3:**
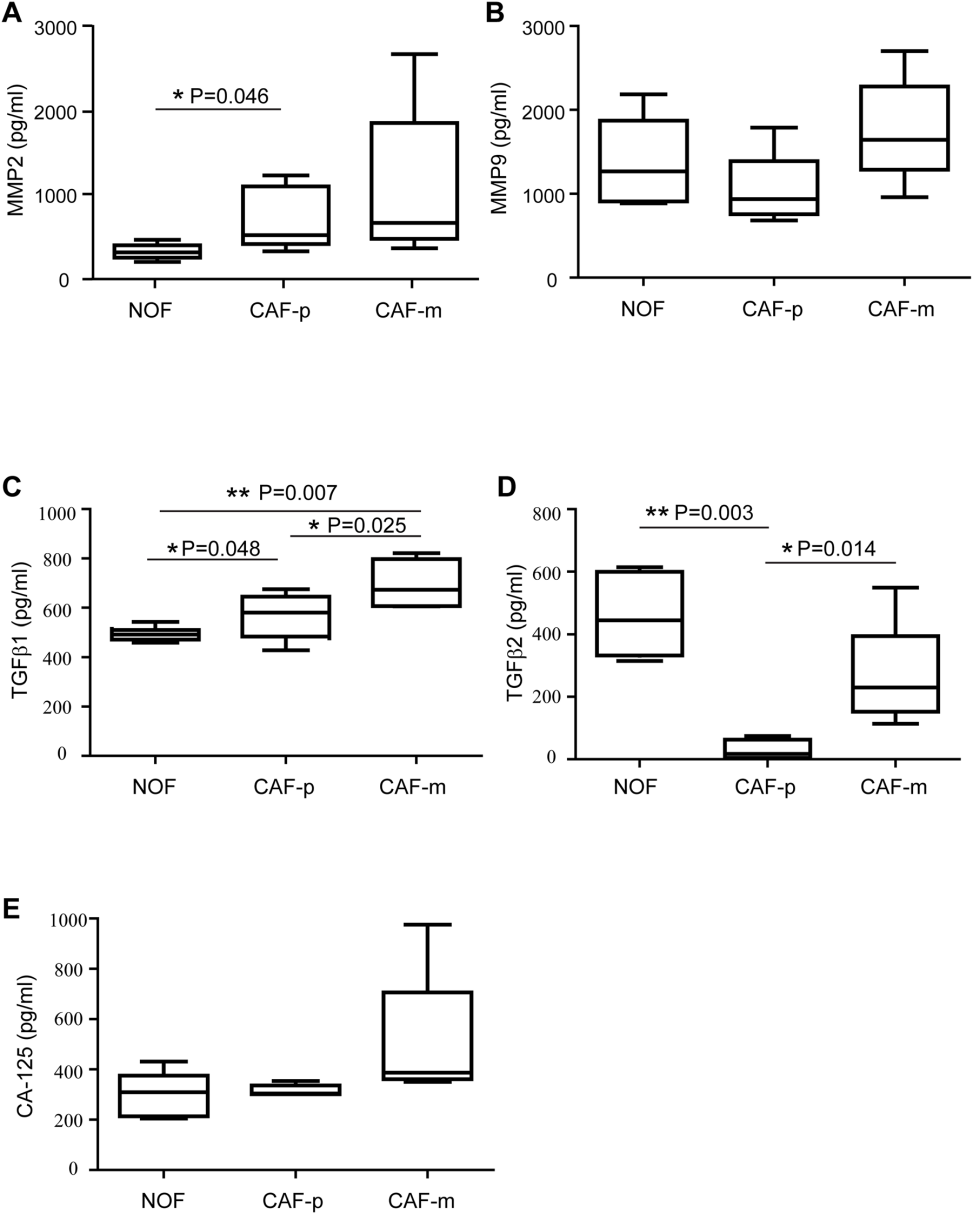
Exosomal expression of MMP2 **(A)**, MMP9 **(B)**, TGFβ1 **(C)**, TGFβ2 **(D)** and CA-125 **(E)** derived from the supernatant of ovarian fibroblasts were quantified by ELISA. The box plots represent the 25th to the 75th quartiles, with the band inside representing the median. The ends of the extended lines indicate the maximum and minimum values. The expression were measured in paired NOF, CAF-p and CAF-m samples from seven high grade serous ovarian cancer patients.

To further understand the proteins secreted by fibroblast exosomes, we detected the concentration of 5 proteins in the exosomes from 7 matched ovarian fibroblast samples (Figure 3). The levels of each protein were compared between the NOF, the CAF-p, and the CAF-m. The expression of TGFβ1 ranged from 502.09 to 967.22 pg/ml, with an average value of 627.71 pg/ml, and a significant upregulation was observed in the CAF-m-derived exosomes compared to the NOF-derived exosomes and those from the CAF-p (Figure 3C). The median increase of TGFβ1 from the NOF to the CAF-m was 1.6-fold, and that from the CAF-p to the CAF-m was 1.4-fold. Interestingly, we noticed that TGFβ2 ranged from 7.73 to 723.55 pg/ml and was notably decreased from the CAF-m to the CAF-p and from the NOF to the CAF-p (Figure 3D). The median decrease from the NOF to the CAF-p was 26.2-fold, and that from the CAF-m to the CAF-p was 13.5-fold.

### Fibroblast-derived TGFβ1 induces EMT in ovarian cancer cells

**Figure 4:**
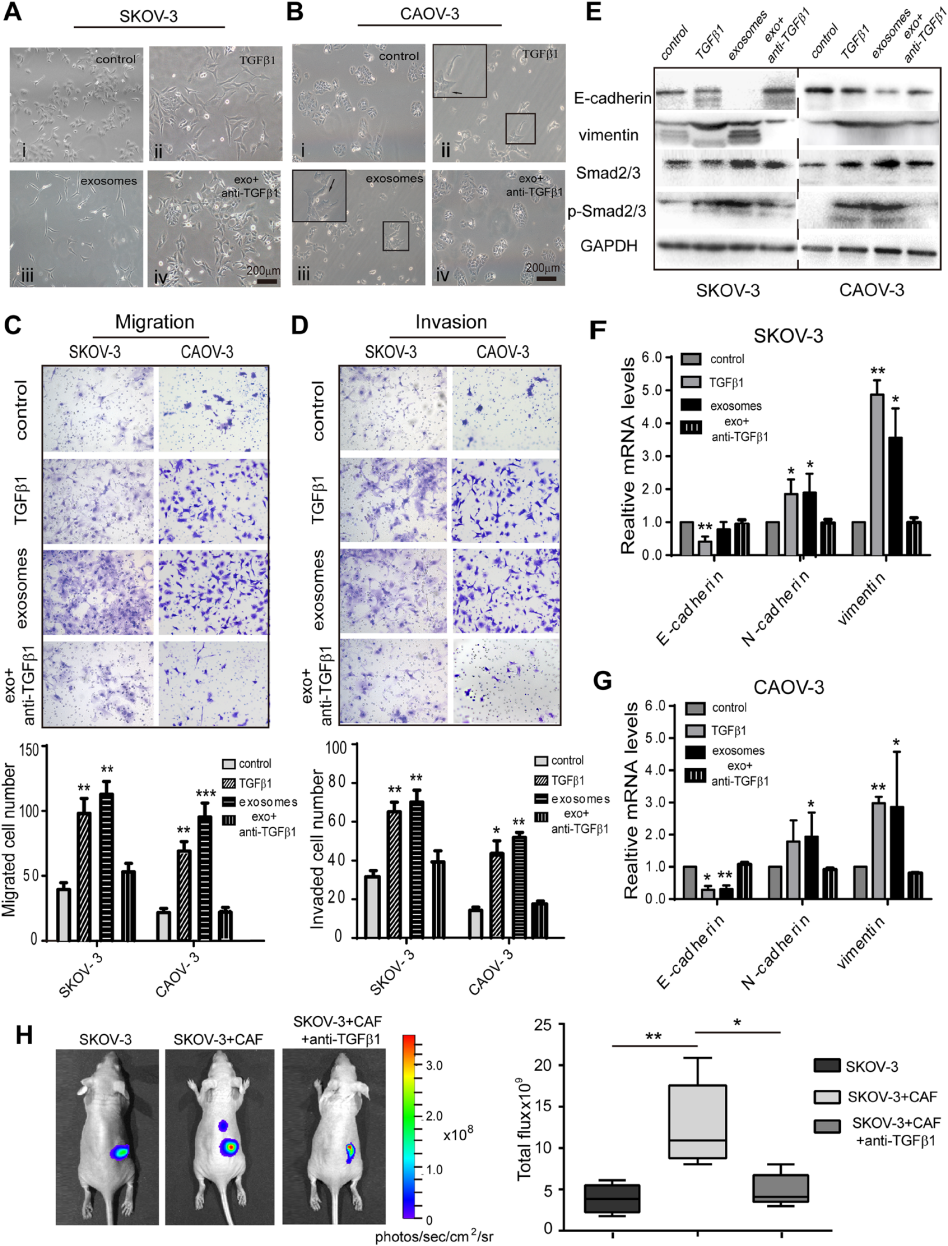
Fibroblast-derived TGFβ1 induces EMT in ovarian cancer cells **(A)** Morphological changes of SKOV-3 cells co-cultured with PBS as control, 10 ng/mL pharmacologic TGFβ1, and CAF-derived exosomes for 72h. Reversal of EMT was formed with TGFβ1 inhibitor treatment. **(B)** Compared with PBS treated control cells, the CAOV-3 co-incubation with fibroblasts exosomes or recombinant TGFβ1 had significantly elongated pseudopodia after 72h. Filopodia formation (arrows) were zoomed in on the upper left panel. Magnification x200. **(C, D)** Cell migration and invasion ability were measured by Transwell assay. Patients CAF-derived exosomes or recombination TGFβ1 induced significantly more migration ability and invasiveness than control group. Data analysis represented on the bottom. **(E)** Ovarian cancer cell lines were stimulated with exosomes or TGFβ1, the expression of EMT markers, E-cadherin and vimentin, and total SMAD2/3, phosphorylation-SMAD2/3 were detected by Western blot. **(F, G)** The expression of EMT-associated transcription factors were detected in the SKOV-3 (F) and CAOV-3 cells (G) by RT-PCR. ^*^ p<0.05, ^**^ p<0.01 and ^***^ p<0.001. **(H)** Representative bioluminescence images of mice (n=5 each group) bearing SKOV-3-Luc cells alone, co-injection with CAF, or with TGFβ1 antibody at 4 weeks after tumor implantation. Bar graph showing the quantification of normalized total photon counts of the subcutaneous xenografts in mice of each group.

To further investigate the role of exosomal TGFβ1 on EMT phenotype changes, we treated the SKOV-3 and CAOV-3 cell lines with recombinant TGFβ1 to assess the effect on the EMT phenotype. Compared to the control group, the pharmacological TGFβ1 demonstrated cellular EMT changes of different degrees. The SKOV-3 formed into an elongated spindle-shaped morphology, and the CAOV-3 cell formed protrusive structures, with cell-cell junction disruptions. This phenomenon was also observed in the CAF-derived exosome-treated group (Figure 4A and 4B, ii and iii). In contrast, using a TGFβ1 blocking antibody reversed the EMT phenotype, resulting in a typical epithelial-like cellular morphology close to the control cells (Figure 4A and 4B, iv). We then measured the migration and invasion capacity of the SKOV-3 and CAOV-3 cell lines after treatment with recombinant TGFβ1. Compared to the control group, the CAF exosome and TGFβ1-treated groups showed significantly enhanced migration and invasion abilities in both cell lines. However, the TGFβ1 neutralizing antibody reduced the migration and invasive capacity of the ovarian cancer cells to levels that were similar to the control (Figure 4C and 4D). Furthermore, treating the SKOV-3 and CAOV-3 cells with exosomes or TGFβ1 increased the expression of mesenchymal markers (N-cadherin and vimentin) yet decreased the epithelial marker E-cadherin at both the protein and mRNA levels compared to the control (Figure 4E–4G). We also examined the phosphorylation levels of SMAD2/3 in ovarian cancer cells after exogenic TGFβ1 stimulation. TGFβ1 and CAF exosomes induced much higher phosphorylation levels of SMAD2/3 in the SKOV-3 and CAOV-3 cells than the control and reversed cells (Figure 4E). These results indicate that exosomal TGFβ1 activates SMAD2/3 signaling to promote an EMT phenotype in ovarian cancer cells.

Due to the interesting decrease in the TGFβ2 levels between the NOF and CAF, we investigated whether TGFβ2 induces EMT in tumor cells as well. We first treated the SKOV-3 and CAOV-3 cells with 10 ng/mL TGFβ2 for 3 days to see if any cell morphology phenotypic changes were induced. Surprisingly, both cells displayed an epithelial shape without obvious signs of an altered mesenchymal phenotype (Supplementary Figure 2A). Then, the migration and invasion abilities of the cells were investigated. As shown in Supplementary Figure 2C, TGFβ2 significantly promoted migration in these two cell lines but not invasion. Another sign of EMT is the down-regulation of E-cadherin and the upregulation of vimentin during TGFβ treatment. The results showed that the expression of E-cadherin protein and mRNA was not altered by TGFβ2, but vimentin was significantly increased in response to TGFβ2 at both the protein and mRNA levels (Supplementary Figure 2B and 2D). Since TGFβ-induced EMT marker variations usually occur via the SMAD signaling pathway, we also examined the phosphorylation levels of SMAD2/3 after exogenic TGFβ2 stimulation. However, no changes were noted when compared to the control and reversed cells (Supplementary Figure 2B).

### Inhibition of TGFβ1 reduces ovarian xenograft tumor growth *in vivo*

To evaluate the importance of TGFβ1 in CAF-derived exosomes supporting a pre-metastatic niche *in vivo*, we mixed SKOV-3-Luc cancer cells and CAF, and co-injected them into BALB/c nude mice, with or without an intraperitoneal injection of anti-TGFβ1. The tumor sizes in the mice injected with the TGFβ1 antagonist were significantly smaller than those in the SKOV-3 with CAF group (Figure 4H). Thus, our data showed that the TGFβ1 in CAF-derived exosomes supports the ovarian cancer cell towards a pre-metastasis state.

### EMT related proteins influenced by fibroblast exosomes

**Figure 5:**
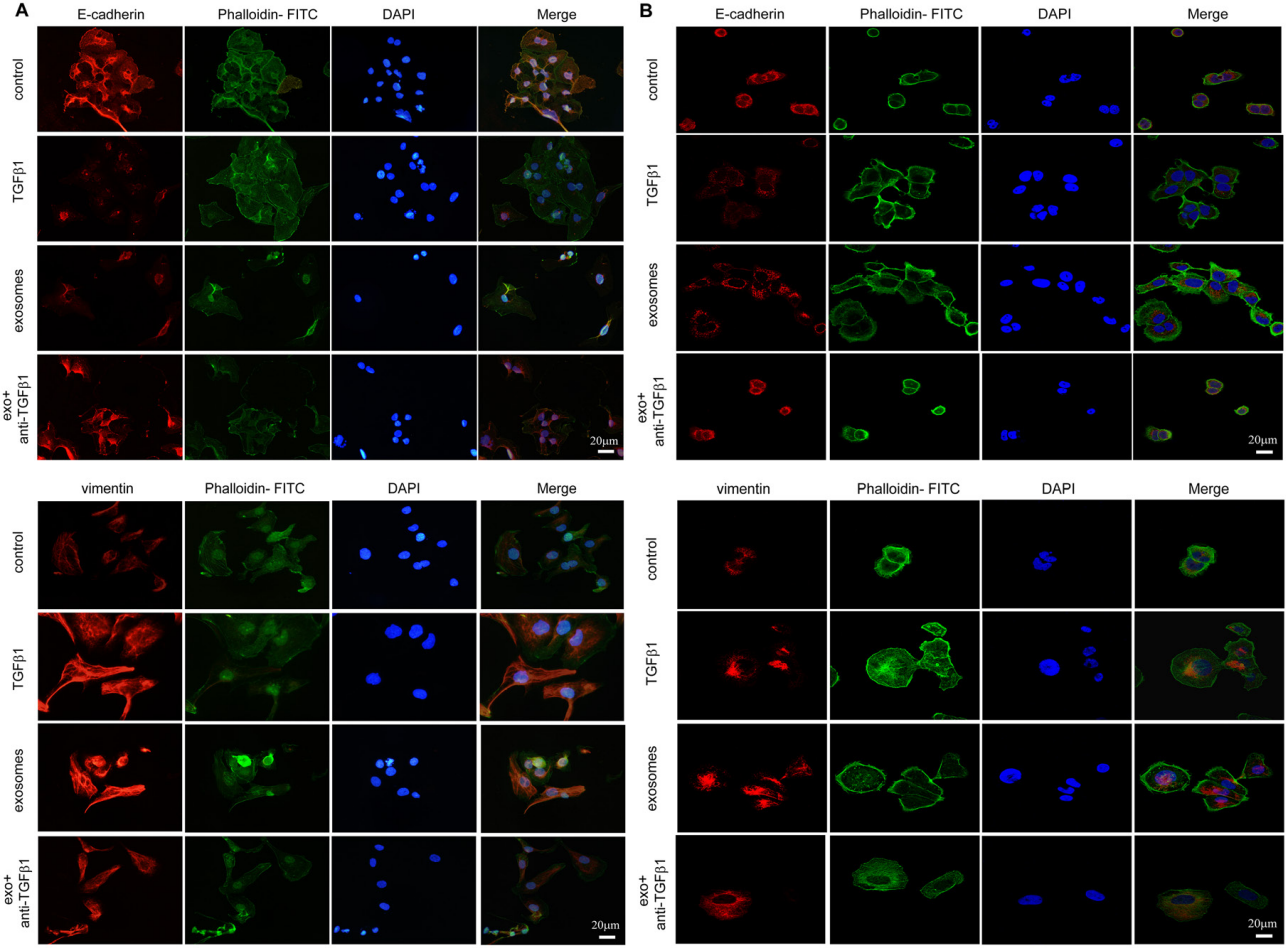
EMT transition of cells cultured with PBS, TGFβ1, CAF-derived exosomes and exosomes with neutralizing TGFβ1 antibody in immunofluorescence **(A)** For SKOV-3 cells, expression of E-cadherin (upper) and Vimentin (lower) were changed during EMT, with F- actin and nuclear DAPI staining demonstrated in merge figures. **(B)** The expression of E-cadherin (upper) and Vimentin (lower) on CAOV-3 cells incubated with different treatments were detected by immunofluorescence staining. Similar variation of protein expressions were observed in both cell lines.

We determined two typical EMT associated proteins, E-cadherin and vimentin, and cellular actin cytoskeletal structure dyed with phalloidin changes during EMT. For SKOV-3 and CAOV-3, fibroblast-derived exosomes induced the F-actin cytoskeletal reorganized markedly, cell-cell junctions lose with E-cadherin expression decreased and vimentin upregulated notably during the transition. Similar actin changes accompanied with EMT markers variation were noticed after pharmacologic TGFβ1 treatment (Figure 5A and 5B).

## DISCUSSION

Metastasis is a major cause of cancer-related mortality in ovarian cancer. The initiation and progression of cancer metastasis is a complex course mediated by tumor cells together with their surrounding microenvironment [8]. CAF, one of the major stromal cell types in ovarian cancer, promotes tumor development, such as migration, invasion, proliferation and metastasis [27, 28]. CAF and tumor cells communicate with each other not only by classical paracrine signaling, for instance, through chemokines, cytokines and growth factors, but also by exchanging bio-information via exosomes. Exosomes are small vesicles derived from endosomes that are secreted locally into other cell types or into body fluids distantly. Therefore, messages from the donor cells are transferred to target cells, which engulf the exosomes, influencing their biological behaviors [13, 29, 30]. Recent studies show that fibroblast-derived exosomes affect the tumor microenvironment to promote cancer cell motility and invasiveness and form a pre-metastatic niche, thereby enabling cancer cell metastasis [31–34]. However, it is still unknown how exosomes from CAF and NOF contribute to epithelial cells during ovarian cancer initiation and metastatic progression.

To clarify this question, we analyzed the protein expression of fibroblast-derived exosomes from different locations of matched ovarian cancer tissue. We showed that TGFβ1 in fibroblast exosomes contributes to EMT in ovarian cancer cells, including the migration and invasion in ovarian epithelial cells, suggesting that CAF-derived exosomes promote ovarian cancer progression and metastasis.

In this study, we first collected the exosomes from ovarian fibroblasts and verified that the fibroblast-derived exosomes were taken up by ovarian tumor cells. Next, we observed that cancer cell migration and invasion increased significantly by CAF-derived exosomes compared to NOF. Furthermore, five proteins related to ovarian cancer progression were selected. As a previous study reported, MMP2 and MMP9 participate in the initiation of omentum metastasis in ovarian cancer. Specifically, MMP2 expression is upregulated when cancer cells are co-cultured with fibroblasts and mesothelial cells [25]. In addition, the role of TGFβ isoforms from the tumor microenvironment play crucial role in EMT [27, 35, 36]. Furthermore, one recent study found that CA-125 induces fibroblasts towards oncogenic transformation [37]. Therefore, we measured these proteins in order to demonstrate how exosomes from the ovarian cancer microenvironment influence neoplastic cells.

**Figure 6:**
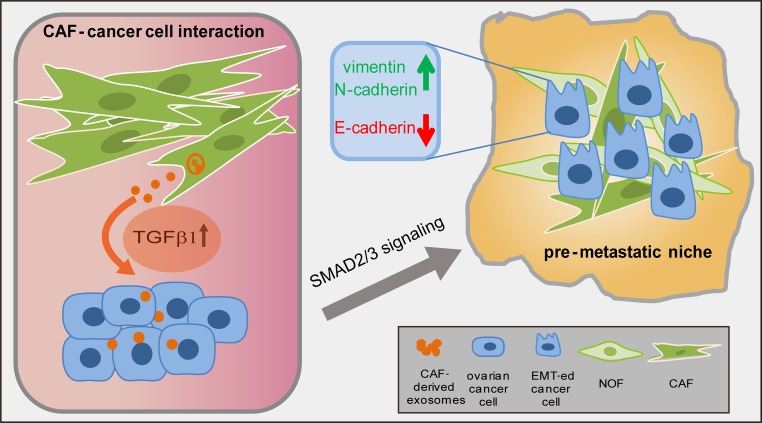
A schematic diagram illustrating the cross-talk of CAF and ovarian cancer cells CAF-derived TGFβ1 transported into cancer epithelial cells and activated the SMAD2/3 signaling to induce EMT, thus promoting tumor peritoneal metastasis.

Our analysis of TGFβ expression showed that CAF were more likely to display higher levels of TGFβ1 but a lower TGFβ2 expression than normal fibroblasts, and both isoforms exhibited an increasing trend from the CAF-p to the CAF-m. The elevated expression of TGFβ1 in the CAF exosomes induced ovarian cancer cell EMT, which was accompanied by a notable decrease in E-cadherin and an increase in vimentin expression (Figure 6). However, TGFβ2 induced ovarian cancer cells into an incomplete or only partial EMT status. Although the role of TGFβ isoforms in tumors remains debatable [38–40], most studies that focus on exosomal TGFβ report that TGFβ1 enhances EMT in diverse cancer models [34–36], and TGFβ2 in milk exosomes drives breast cancer cells towards EMT [27]. However, low levels of TGFβ2 were reported to promote lung metastasis in head and neck squamous carcinoma [41]. Previous studies showed that TGFβ1 and TGFβ2 were both capable of inducing complete EMT in OVCA429, a clear-cell ovarian cancer cell line [20], but a partial EMT was observed in OVCAR3, a serous ovarian cell [38]. Similarly, in our study, TGFβ2 promoted the SKOV-3 and CAOV-3 cells to an uncomplete EMT phenotype, which may also be because we chose two serous cancer cell lines, the most common histologic subtype in ovarian cancer. The subtype differences could possibly explain this interesting outcome. For the upregulation from primary to metastasis fibroblasts by TGFβ1 and TGFβ2, this may be because the role of TGFβ is strongly context-dependent in the metastatic progress, including cell and cancer type [42]. Meanwhile, during cancer progression, increased expression of TGFβ is associated with metastasis, which induces fibroblast adjacent to tumor cells activation upon tumor stages [43]. Our data supports the above findings, and suggest that futher studies should consider the role of TGFβ in ovarian cancer EMT depending on various histologic subtypes and tumor anatomic locations.

Our results showed that CAF express significantly higher TGFβ1 levels than normal fibroblasts, and the transfer of exosomes from CAF to neighboring cancer cells promotes EMT in ovarian cancer, which thus initiates tumor progression and metastasis. Our study presents a new potential role for CAF-derived exosomes and their importance in cancer progression.

In conclusion, our findings implicate that CAF-derived exosomes are involved in the crosstalk between the ovarian microenvironment and epithelial cells. We demonstrated that TGFβ1 is enriched in CAF exosomes, which are transferred to ovarian tumor cells, inducing EMT through SMAD signaling. Our findings provide an important view for cancer therapy that focuses on stromal-derived exosomes, suggesting that inhibiting or preventing the transfer of exosomes from CAF may be a new strategy for suppressing ovarian cancer progression and metastasis.

## MATERIALS AND METHODS

### Cell lines and primary fibroblast samples

SKOV-3 and CAOV-3 cells were purchased from ATCC (American Type Culture Collection). SKOV-3 was cultured in McCoy's 5A and CAOV-3 in DMEM medium supplemented with 10% FBS and 1% penicillin-streptomycin at 37°C with 5% CO_2_. These cell lines were verified by the source organization before purchased, confirmed no mycoplasma contamination and used in 3 months after recovery from frozen aliquot.

Patient samples from ovarian primary cancer-associated fibroblasts (CAF-p), matched CAF from omental metastasis (CAF-m), and normal omental fibroblasts (NOF) at least 5 cm from the metastasis tumor margin were isolated as previously described [9, 44]. The sample tissues used for the isolation were first affirmed by at least two senior pathologists as high-grade serous ovarian carcinoma, and then, the fresh surgical specimens were transferred on ice immediately. The fibroblasts were separated within 2 hours after the collection. Due to the large amount of cells needed to gather exosomes and our strict matching enrollment standard in this paper, we collected more than 20 specimens during the past two years from Tongji Hospital, Huazhong University of Science and Technology, and seven matched pairs of the three lesions fibroblasts were obtained for sufficient exosomes for the following experiment (Supplementary Figure 1A and 1B). All the fibroblasts were grown in DMEM/F-12 medium and were used between passage 3 and 6. To confirm that the exosomes derived from the surgical specimens were pure fibroblasts, we used vimentin, α-SMA, E-cadherin and cytokeratin 8+18 to exclude epithelial cell contamination (Supplementary Figure 1C–1F). This study was approved by the Ethics/Institutional review board of Tongji Hospital, Wuhan, China. Written informed consent according to the Declaration of Helsinki was obtained from all seven patients.

### Exosomes isolation and labeling

The exosomes from the fibroblasts were isolated using the serial centrifugation method as previously described [11]. When the human fibroblasts reached 80-90% confluency, the cells were washed three times with PBS and were incubated in serum-free medium for 24- 48 hours before exosome collection. The medium was centrifuged at 300 g for 5 min, 2000 g for 10 min, and 10,000 g for 30 min, filtered through a 0.22 μm filter and was ultra-centrifuged again at 110,000 g for 70 min followed by another 110,000 g for 70 min. The exosome pellets were resuspended in 100 μl of phosphate buffered saline (PBS) and were stored at -80°C until further analyses. The purified exosome pellets were labeled using the Dil dye (Sigma) according to the manufacturer's instructions. The SKOV-3 and CAOV-3 cells were co-cultured with fibroblast supernatant exosomes for 6 h. Images were taken using a fluorescence microscope to confirm the presence of exosomes within the cells.

### Transmission electron microscopy (TEM)

Exosomes were placed onto the formvar-carbon-coated TEM grids. Samples were negatively stained with 1% glutaraldehyde for 5 min, fixed in a mixture of 4% uranyl acetate and 2% methyl cellulose for 5 min at room temperature. The samples were then visualized by a Tecnai G20 TWIN operated at 200kV (FEI Company, Oregon, USA).

### NanoSight particle tracking analysis

We analysed the purified exosomes by the NanoSight LM10 system (NanoSight Malvern, UK) as described before [18]. Briefly, exosomes were diluted in PBS, then applied to a blue laser beam at 405 nm. NanoSight tracking analysis (NTA) software analyzed the samples and calculated exosomes particle size and concentration.

### Western blot analysis

The cells and exosomes were lysed in radio immunoprecipitation assay (RIPA) buffer. Protein concentration was measured by the BCA Protein Assay kit following the manufacturer's instructions (Thermo Scientific Pierce). 30μg protein was loaded for each sample on a 12% SDS-PAGE gel, and then transferred to a PVDF membrane (Millipore, MA, USA) that was pretreated with methanol. The membrane was blocked, rinsed, and subsequently incubated with primary antibodies against CD-63 (Santa Cruz), α-SMA, vimentin, E-cadherin, TSG101 and Smad 2/3 (Abcam), and p-Smad2 (Ser 465/467)/Smad3 (Ser 423/425) (Cell Signaling Technology) overnight at 4°C. After vigorous washing, the blots were incubated with their corresponding secondary antibodies. The labeled proteins were detected by an enhanced chemiluminescence (ECL) kit (Amersham Biosciences, Buckinghamshire, UK).

### Enyzme-linked immunosorbent assay (ELISA)

The expression of 5 proteins (MMP2 and 9, TGFβ1 and 2 and CA-125) in the fibroblast exosomes was measured in duplicate by ELISA. The MMP9, TGFβ1 and 2 ELISA kits (eBioscience, Minneapolis, MN) were used according to the manufacturer's instructions. The MMP2 ELISA kit (Abcam, Cambridge, MA) and the CA-125 ELISA kit (Boster, Wuhan, China) were used as well. Prior to ELISA, the exosomes were treated with 20 μg/mL of proteinase K (Boster, Wuhan, China) at 55°C for 10 min to dissolve the membrane-associated proteins. Then, the exosome pellet was resuspended in dilute buffer and was added to each well. Sample absorbance was measured using a plate reader at 450 nm, following the manufacturer's instructions.

### EMT

The SKOV-3 and CAOV-3 cells were washed with PBS twice, and 2×10^5^ cells were co-incubated with the fibroblast exosomes (20 μg) and with 10 ng/ml of human recombinant TGFβ1 or TGFβ2 (PeproTech EC Ltd., UK) for 72 h, with or without 10 μg TGFβ1 and 10 μg TGFβ2 blocking antibodies (R&D Systems). Cell morphology was then visualized by an inverted light microscopy.

### Immunofluorescence

The SKOV-3 and CAOV-3 cells were grown on cover slips and were fixed with 4% paraformaldehyde, washed with PBS twice, permeabilized with 0.1% TritonX-100, washed again and blocked with 1% BSA Then, the samples were incubated with the primary antibody overnight at 4°C. The antibodies included α-SMA, vimentin and cytokeratin 8+18 (1:100, Abcam) and E-cadherin (1:200, Abcam). After washing with PBS, the cells were incubated with Alexa Fluor 549 (1:1000, Invitrogen), FITC phalloidin for F-actin (Life technologies) and DAPI for nuclear staining (Sigma). The fluorescence images were photographed with an Olympus BX53 microscope (Olympus).

### Flow cytometry

The fibroblast samples were incubated for 30 minutes with the following mouse anti-human monoclonal antibodies: anti-cytokeratin 8+18 (1:500, R&D Systems) and anti-α-SMA (1:200, Abcam). Then, the samples were stained with an anti-mouse PE-labeled secondary antibody (R&D Systems). The samples were acquired using a fluorescence activating cell sorter (FACS) Calibur flow cytometer (BD Biosciences). The flow cytometry data were analyzed using FlowJo software (Tree Star, Ashland, OR).

### Quantitative real time (RT)-PCR

The mRNA expression levels were determined by real-time RT-PCR as previously described [43]. Briefly, total RNA was extracted from the cells, and 2 μg of total RNA was reverse transcribed to cDNA. The RT-PCR reactions were performed using the IQ SYBR Green supermix (Bio-Rad, Hercules, CA) according to the manufacturer's instructions. The relative fold changes in gene expression were calculated using the 2−ΔΔCT formula. GAPDH mRNA levels served as the internal control. The expression levels of E-cadherin, vimentin, and N-cadherin after treating the SKOV-3 and CAOV-3 cells with the negative control and exosomes, as well as the TGFβ1 and TGFβ2, were performed in triplicate. The primers used in this study are the following: E-cadherin, 5'-CCCACCACGTACAAGGGTC-3' (forward), 5'-CTG GGGTATTGGGGGCATC-3' (reverse); vimentin, 5'-ACT ACGTCCACCCGCACCTA-3' (forward), 5'-CAGCG AGAAGTCCACCGAGT-3' (reverse); N-cadherin, 5'-TCAGGCGTCTGTAGAGGCTT-3' (forward), 5'-ATG CACATCCTTCGATAAGACTG-3' (reverse); GAPDH, 5'-ACAACTTTGGTATCGTGGAAGG-3' (forward), 5'-GCCATCACGCCACAGTTTC-3' (reverse).

### Transwell migration and invasion assays

For ovarian cancer cell migration and invasion, 1×10^5^ cells/mL were seeded in serum-free media onto 24-well Transwell inserts with 8.0 μm PET membranes (Corning). Media containing 1% FBS served as the control group, with 10 μg exosomes or recombinant TGFβ1 added to the bottom wells. The migrated cells (at 24 hours) or the invaded cells (at 48 hours) were fixed and stained with 0.1% crystal violet (Sigma-Aldrich). Images from five random fields (200× magnification) were taken, and the number of cells in each chamber was counted. The experiments were performed in triplicate wells using exosomes from 3 different patients, and each experiment was performed at least three times as indicated.

### Wound-healing assay

The SKOV-3 and CAOV-3 cells were seeded in 6-well plates to achieve confluence, wounds were scratched using a 200 μl micropipette and cultures were washed with PBS to remove detached cells. The cell cultures were carried out in serum-free medium with or without 20 μg exosomes and photographed at 0 and 12 h. The relative closure rate of each sample was measured using ImageJ software.

### Animal studies

The animal experiments were performed under the guidelines of the Committee on the Ethics of Animal Experiments in Hubei Province. Six-week-old female BABL/c nude mice (Huafukang, Beijing, China) were used in the experiments. The SKOV-3 cells were stably transduced with CMV-Fluc-IRES-RFP lentiviral particles (GeneChem, shanghai, China) and previously designated SKOV-3-Luc. The orthotopic ovarian cancer mouse model was established by injecting the SKOV-3-Luc cell (2×10^6^ cells in 25 μl of PBS) either alone or co-injected with 4×10^6^ CAF into the right ovary (n=5 per group). Three days later, PBS or 10 μg anti-TGFβ1 was injected into the abdominal cavity every other day six times. Four weeks later, the photons were measured by assessing bioluminescence via *in vivo* imaging using an IVIS Spectrum system (Caliper Life Science, Xenogen, MA) ten minutes after an intraperitoneal injection of D-luciferin (Promega, Madison, WI). The total flux (photons/s) of the xenografts was analyzed using the Living Image version 4.3.1 software.

### Statistical analysis

The statistical analysis was performed using GraphPad Prism 5.0 software. The data are presented as the mean ±SEM. The association between two groups was determined using a two-tailed Student's *t* test. The comparisons among multiple groups were analyzed by a one-way ANOVA, and Tukey's post-test was further used. P values < 0.05 were considered statistically significant.

## SUPPLEMENTARY MATERIALS FIGURES


