# Supplementary material for: TGFβ1 in fibroblasts-derived exosomes promotes epithelial-mesenchymal transition of ovarian cancer cells
Source: Oncotarget. 2017 Oct 6;8(56):96035–47. doi: 10.18632/oncotarget.21635 (PMC5707079; doi:10.18632/oncotarget.21635)
Supplement: Supplementary file 1 [file oncotarget-08-96035-s001.pdf]

TGFβ1 in fibroblasts-derived exosomes promotes epithelial-mesenchymal transition of ovarian cancer cells

SUPPLEMENTARY MATERIALS

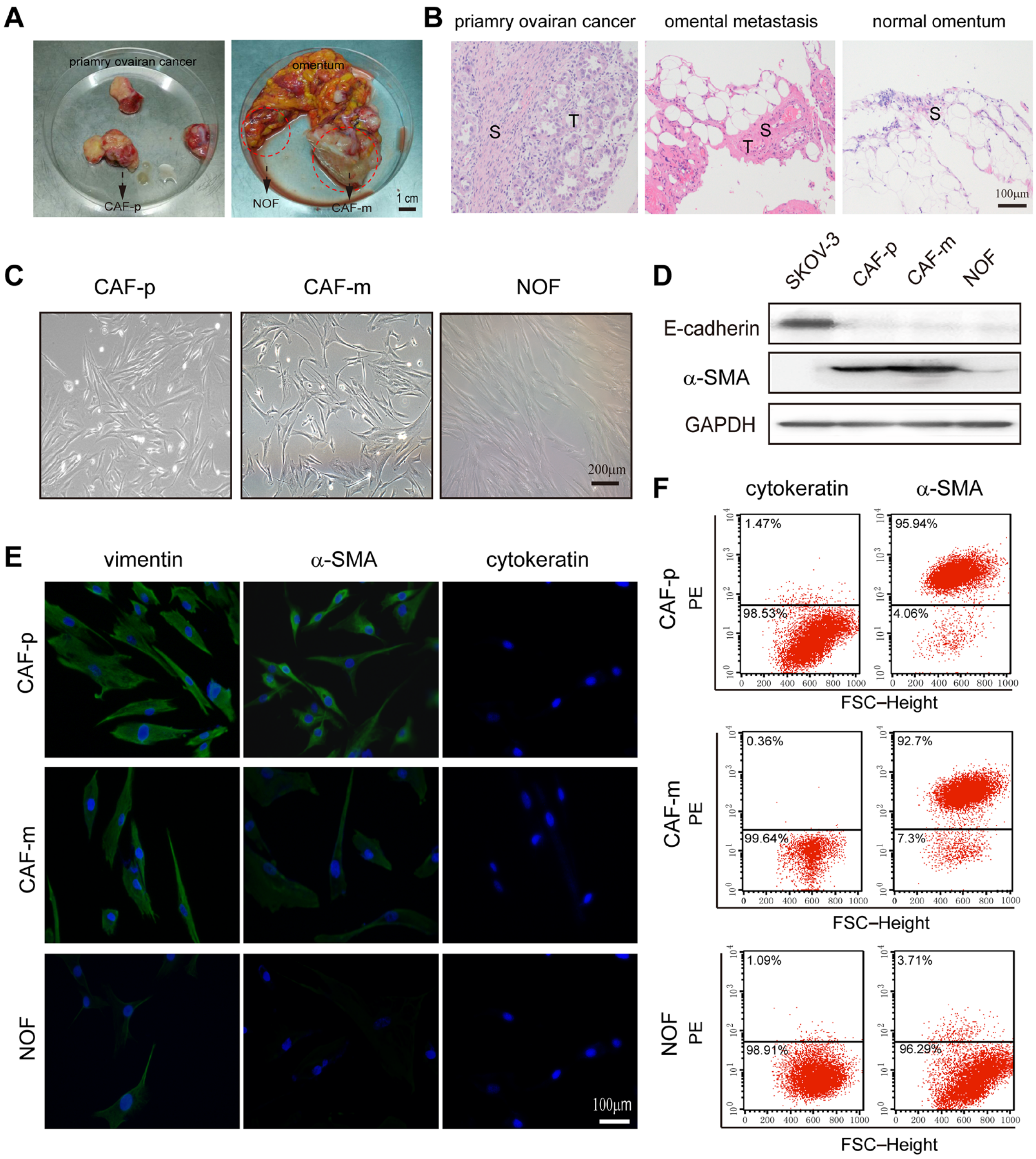

Supplementary Figure 1: Characterization of fibroblasts isolated from ovarian cancer patients. **(A)** Left, typical appearance of primary ovarian carcinoma tissue placed in cell culture dish before CAF-p cultured. Right, isolation of CAF-m and metastatic tumor adjacent NOF from ometum of serous ovarian cancer patients. **(B)** Representative H&E images of paraffin-embedded sections from matching primary ovarian cancer tissue, omental metastatic tissue and normal omentum (T, tumor; S, stroma). **(C)** Morphological images of cultured ovarian CAF-p, CAF-m and NOF between 3 to 6 passages. **(D)** Immunoblotting of E-cadherin and vimentin in stromal fibroblasts isolated from different ovarian cancer tissues. GAPDH was used as a loading control. **(E)** Immunofluorescence staining (green) of vimentin, α-smooth muscle actin (α-SMA) and cytokeratin 8+18 in CAF-p, CAF-m and NOF. The cell nucleus were stained with DAPI (blue). **(F)** Flow cytometry indicating positive α-SMA expression in CAF-p and CAF-m while negative in NOF, but negative cytokeratin expression in both CAF and NOF.

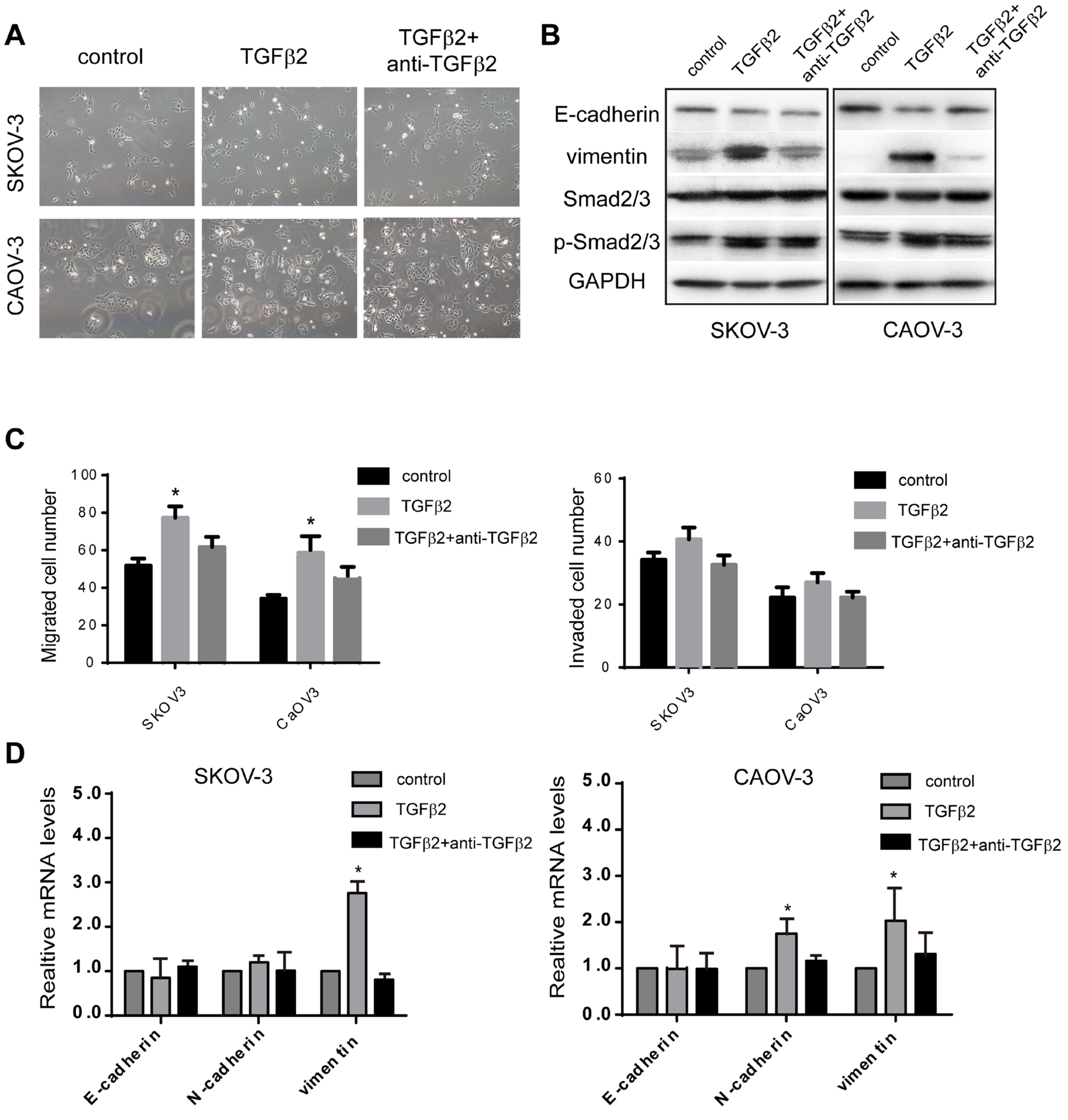

Supplementary Figure 2: Effect of TGFβ2 on ovarian epithelial cells. **(A)** Morphological changes of SKOV-3and CAOV-3 cells co-cultured with PBS as control, 10 ng/mL pharmacologic TGFβ2 with or without 10 μg/ml anti-TGFβ2 for 72h. **(B)** Ovarian cancer cell lines were stimulated with TGFβ2, the expression of EMT markers, E-cadherin and vimentin, and total SMAD2/3, phosphorylation-SMAD2/3 were detected by Western blot. **(C)** Cell migration and invasion ability were measured by Transwell assay. **(D)** The expression of EMT-associated transcription factors were detected in the SKOV-3 and CAOV-3 cells by RT-PCR. * $p<0.05$.
